# Investigation of ComBat Harmonization on Radiomic and Deep Features from Multi-Center Abdominal MRI Data

**DOI:** 10.1007/s10278-024-01253-0

**Published:** 2024-09-16

**Authors:** Wei Jia, Hailong Li, Redha Ali, Krishna P. Shanbhogue, William R. Masch, Anum Aslam, David T. Harris, Scott B. Reeder, Jonathan R. Dillman, Lili He

**Affiliations:** 1 Imaging Research Center, Department of Radiology, Cincinnati Children’s Hospital Medical Center, 3333 Burnet Avenue, MLC 7009, Cincinnati, OH 45229 USA; 2 Department of Environmental and Public Health, Division of Biostatistics and Bioinformatics, University of Cincinnati, Cincinnati College of Medicine, Cincinnati, OH USA; 3 Department of Radiology, University of Cincinnati College of Medicine, Cincinnati, OH USA; 4 Department of Radiology, New York University Langone Health, New York, NY USA; 5 Department of Radiology, University of Michigan, Michigan Medicine, Ann Arbor, MI USA; 6 Departments of Radiology, Medical Physics, Biomedical Engineering, Medicine, Emergency Medicine, University of Wisconsin, Madison, WI USA; 7 Computer Science, Biomedical Engineering, Biomedical Informatics, University of Cincinnati, Cincinnati, OH USA

**Keywords:** Multi-center study, ComBat, Harmonization, Radiomics, Deep learning, Artificial intelligence, Magnetic resonance imaging

## Abstract

ComBat harmonization has been developed to remove non-biological variations for data in multi-center research applying artificial intelligence (AI). We investigated the effectiveness of ComBat harmonization on radiomic and deep features extracted from large, multi-center abdominal MRI data. A retrospective study was conducted on T2-weighted (T2W) abdominal MRI data retrieved from individual patients with suspected or known chronic liver disease at three study sites. MRI data were acquired using systems from three manufacturers and two field strengths. Radiomic features and deep features were extracted using the PyRadiomics pipeline and a Swin Transformer. ComBat was used to harmonize radiomic and deep features across different manufacturers and field strengths. Student’s *t*-test, ANOVA test, and Cohen’s F score were applied to assess the difference in individual features before and after ComBat harmonization. Between two field strengths, 76.7%, 52.9%, and 26.7% of radiomic features, and 89.0%, 56.5%, and 0.1% of deep features from three manufacturers were significantly different. Among the three manufacturers, 90.1% and 75.0% of radiomic features and 89.3% and 84.1% of deep features from two field strengths were significantly different. After ComBat harmonization, there were no significant differences in radiomic and deep features among manufacturers or field strengths based on *t-*tests or ANOVA tests. Reduced Cohen’s F scores were consistently observed after ComBat harmonization. ComBat harmonization effectively harmonizes radiomic and deep features by removing the non-biological variations due to system manufacturers and/or field strengths in large multi-center clinical abdominal MRI datasets.

## Introduction

Magnetic resonance imaging (MRI) has emerged as a valuable tool for diagnostic evaluation of the abdomen, offering unprecedented contrast resolution without ionizing radiation [1]. This imaging modality is routinely used to noninvasively visualize various organs and tissues, including (but not limited to) the liver, spleen, pancreas, kidneys, and bowel among others [2–4].

Radiomic features are hand-crafted, mathematically defined quantitative features that are used to quantify the characteristics of organs and lesions, such as morphology, signal intensity, and texture of medical images [5]. Such features have been widely used in medical research and clinical practice for diagnostic, prognostic, and treatment planning purposes [6–9]. Deep features are high-dimensional feature representations that are automatically learned by deep learning models [10]. The hierarchical nature of deep learning models allows them to capture increasingly abstract information as images pass through the model, enabling the automatic extraction of meaningful and discriminative deep features from complex MRI data [11–15]. Both radiomic and deep features have been increasingly applied to abdominal MR image analyses [5, 7, 10, 16–18].

There is increasing interest in multi-center research applying artificial intelligence (AI) techniques to abdominal MRI data due to several advantages over single-center investigations, including a larger sample size, increased statistical power, and improved model generalizability and reliability [19, 20]. However, multi-center studies using clinical abdominal MRI data can suffer from non-biological variations due to differences in image acquisition protocols, field strength, or system manufacturers, either within or between different sites [21, 22]. These non-biological variations, often referred to as batch effects [23], may result in poor reproducibility of image features extracted from MR images [24]. Data harmonization techniques are essential to mitigate the batch effects by standardizing MRI data acquired from different systems or imaging protocols [22, 23]. Initial harmonization methods have focused on preprocessing methods, such as intensity normalization and denoising [6, 25]. However, studies have shown that the effectiveness of such preprocessing methods is very limited [24].

ComBat harmonization [26] has been developed to remove non-biological variations and ensure the consistency of features. Originally proposed for genomic studies, ComBat is a statistical method that utilizes an empirical Bayesian framework to adjust batch effects in the data. By assuming that the observed data follow a multivariate normal distribution, the ComBat method decomposes data into two components: a biological signal of interest and batch-specific effects. It then adjusts the data by removing batch effects while minimizing changes in the biological signal. A few previous studies have demonstrated increased robustness of radiomic features after ComBat harmonization using relatively small cohorts [27, 28].

In this study, we aim to investigate the effectiveness of ComBat harmonization on both radiomic and deep features using clinical abdominal MRI data from a large multi-center adult cohort. Specifically, we focus on both radiomic and deep features extracted from T2-weighted (T2W) clinical abdominal MR images. We seek to test the hypothesis that ComBat can effectively harmonize radiomic and deep features from MRI systems of different field strengths and manufacturers.

## Materials and Methods

The Institutional Review Boards of three geographically dispersed institutions, including Site 1, Site 2, and Site 3, approved this multi-center retrospective study. We performed this study in accordance with the ethical standards described in the 1964 Declaration of Helsinki and its later amendments. Patient informed consent was waived. All patient health information (PHI) was de-identified prior to data sharing. All study activities complied with the Health Insurance Portability and Accountability Act (HIPAA).

### Multi-Center Study Cohort

From three academic medical centers, we identified adult patients (i) with suspected or known chronic liver disease and (ii) who underwent MRI examinations between 2011 and 2022. We collated and anonymized consecutive clinical abdominal T2-weighted (T2W) fast spin-echo fat-saturated MRI datasets from clinical Picture Archiving and Communicating Systems (PACS). Abdominal MRI examinations were performed on 1.5 Tesla (T) and 3 T MRI systems manufactured by Siemens (Siemens Healthineers, Erlangen, Germany) for Site 1, GE (GE HealthCare, Chicago, IL, USA) for Site 2, and Philips (Philips Healthcare, Amsterdam, Netherlands) for Site 3.

### Extraction of Radiomic Features

To extract radiomic features, we used a previously trained Swin U-Net Transformer (Swin UNetR) [29] to segment both the liver and spleen from all T2W MR images to accelerate organ segmentation from our large, multi-center cohort. Swin UNetR [29] is a U-shaped encoder-decoder structured network with a Swin Transformer as the encoder and a convolutional neural network as the decoder. Next, we used the PyRadiomics [30], a Python library pipeline, to extract first-order radiomic features, gray level co-occurrence matrix features, gray level size zone matrix features, gray level run length matrix features, and gray level dependence matrix features from segmented liver and spleen. Morphology-based (i.e., size and shape) features were excluded from our analysis since those features are irrelevant to batch effects. We extracted 86 radiomic features for the liver and spleen, separately. This resulted in a total of 172 radiomic features for each MRI exam.

### Extraction of Deep Features

To extract deep features, we adopted a Swin Transformer [31] as the feature extractor for its superior image recognition performance [29, 32, 33]. Swin Transformer achieves deep feature extraction by using a shifted window hierarchical architecture that efficiently processes high-resolution images. Specifically, we used a Swin transformer model that was trained using the ImageNet dataset (~ 1.2 million natural color images) [31]. Eleven unsegmented T2W images that can cover the central liver and spleen were selected from each T2W MRI exam. Individual two-dimensional (2D) axial images were input to the Swin transformer, generating 1024 deep features for each MR image. We applied a global average pooling layer after Swin Transformer to combine deep features from all 2D images, resulting in a total of 1024 deep features for each MRI exam.

### ComBat Harmonization

Assuming that the batch effects follow a normal distribution, the ComBat harmonization method [26] first fits a linear model to estimate the batch effects within the data and then subtracts the estimated batch effects from the original data. Specifically, we denoted the numerical value of the radiomic or deep feature $f$ of the subject $j$ at the site $i$ as ${Y}_{ijf}$, which can be modeled as

$${Y}_{ijf}={\alpha }_{f}+{\gamma }_{if}+X{\beta }_{f}+{\delta }_{if}{\epsilon }_{ijf}$$

where ${\alpha }_{f}$ is the mean of the feature $f$, ${\gamma }_{if}$ is the additive batch effect, ${\delta }_{if}$ is the multiplicative batch effect, and ${\beta }_{f}$ is the regression coefficients of covariates. The covariate matrix $X$ contains two common covariates for MRI data, i.e., age at the time of MR imaging and patient sex. The error term ${\epsilon }_{ijf}$ is assumed to follow a normal distribution. Then, we calculated the harmonized radiomic or deep feature as

$${Y}_{ijf}^{*}=\frac{{Y}_{ijf}-\widehat{{\alpha }_{f}}-\widehat{{\gamma }_{if}}-X\widehat{{\beta }_{f}}}{\widehat{{\delta }_{if}}}+\widehat{{\alpha }_{f}}+X\widehat{{\beta }_{f}}$$

where $\widehat{{\alpha }_{f}}$, $\widehat{{\gamma }_{if}}$, $\widehat{{\delta }_{if}}$, and $\widehat{{\beta }_{f}}$ are estimators of ${\alpha }_{f}$, ${\gamma }_{if}$, ${\delta }_{if}$, and ${\beta }_{f}$, respectively.

### Statistical Analysis

**Fig. 1:**
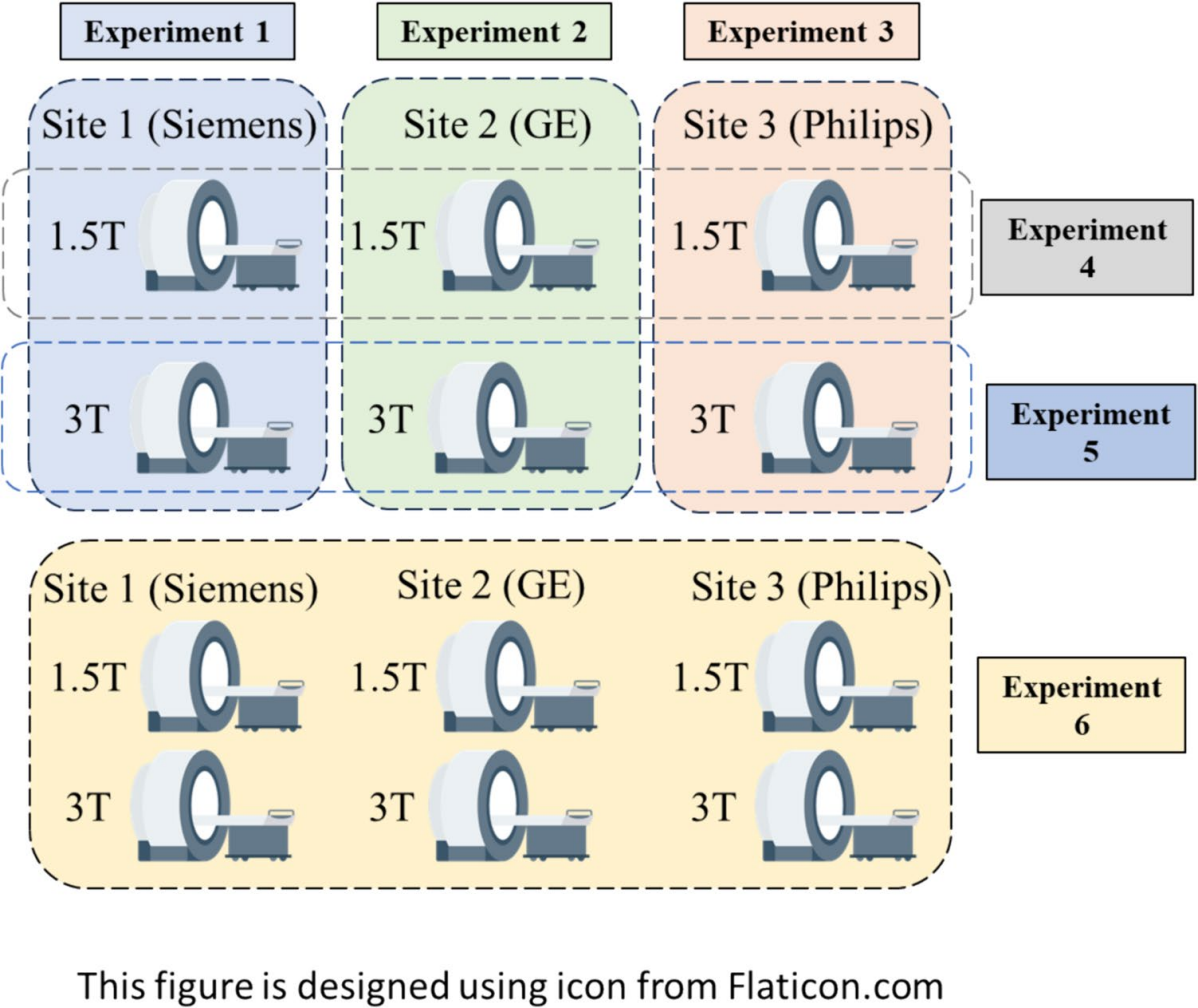
Study design to test the effectiveness of ComBat harmonization. Experiments 1–3 were designed to test whether ComBat can effectively harmonize features from the same manufacturers but different field strengths. Experiments 4–5 were designed to test whether ComBat can effectively harmonize features from the same field strength, but different manufacturers. Experiment 6 was utilized to assess whether the ComBat can harmonize features from MRI systems of different manufacturers and field strengths. This figure is designed using an icon from Flaticon.com

To test our hypothesis, we designed six separate experiments for both radiomic and deep features, respectively (Fig. 1). Experiments 1–3 were designed to test whether ComBat can effectively harmonize features from the same manufacturer but different field strengths (i.e., Site 1 (Siemens) 1.5 T vs 3 T; Site 2 (GE) 1.5 T vs 3 T; Site 3 (Philips) 1.5 T vs 3 T). Experiments 4–5 were designed to test whether ComBat can effectively harmonize features from the same field strength but different manufacturers (i.e., 1.5 T MRI systems – Site 1 (Siemens) vs Site 2 (GE) vs Site 3 (Philips), and 3 T MRI systems – Site 1 (Siemens) vs Site 2 (GE) vs Site 3 (Philips)). Experiment 6 was utilized to assess whether ComBat can harmonize features from all different field strengths and manufacturers.

We applied the student’s *t*-test or ANOVA test for each individual radiomic or deep feature with the null hypothesis that there is no difference among feature distributions from different field strengths and/or manufacturers. Cohen’s F score [34] was used as a measure of effect size in *t*-test/ANOVA for individual features as below:

$${\eta }_{p}^{2}=\frac{S{S}_{effect}}{S{S}_{effect}+S{S}_{error}}$$

$$Cohe{n}^{\prime}s \;F=\sqrt{\frac{{\eta }_{p}^{2}}{1-{\eta }_{p}^{2}}}$$

A larger Cohen’s F score indicates a larger difference between feature distributions, suggesting that there are large batch effects between features from different systems. To control the family-wise error rate in the context of multiple comparisons, we employed the Bonferroni correction. An adjusted *p*-value < 0.05 was considered significant in the current study. We used the chi-square test to compare patient sex and the ANOVA test to compare patient age between study sites. All statistical analyses were performed with R (version 4.3.1) [35].

## Results

### Demographic Characteristics of Multi-Center Study Sample

We included 3629 unique adult patients in this multi-site study. The study included participants from three different sites. At Site 1, MRI exams were acquired from 2076 patients with a mean ± standard deviation (SD) age of 53.8 ± 13.9 years, of whom 1076 (51.8%) were female. At Site 2, MRI examinations were acquired from 1226 patients with a mean ± SD age of 51.5 ± 15.4 years, of whom 563 (50.4%) were female. At Site 3, MRI examinations were acquired from a total of 327 patients with a mean ± SD age of 54.9 ± 13.8 years, of whom 174 (53.2%) were female. Additional demographic information is listed in Table 1. No significant difference was found in age (*p* = 0.23) or sex (*p* = 0.47) between the three sites. A total of 3857 examinations were retrieved. Among them, 2304 were from Site 1, 1226 were from Site 2, and 327 were from Site 3.

**Table 1:** Detailed demographics of the cohort from three study sites

Site	Number of MRI scans	Sex: female (ratio)	Age: mean ± STD	System manufacturer	Field strength
Site 1	1614	837 (52%)	53.6 ± 14.1	Siemens	1.5 T
Site 1	690	364 (53%)	53.8 ± 13.4	Siemens	3 T
Site 2	427	214 (50%)	50.7 ± 15.2	GE	1.5 T
Site 2	799	402 (50%)	51.9 ± 15.5	GE	3 T
Site 3	315	166 (53%)	54.9 ± 13.8	Philips	1.5 T
Site 3	12	8 (50%)	54.2 ± 14.9	Philips	3 T

### Effectiveness of ComBat Harmonization on Radiomic Features

Table 2 shows the number of features with significant differences and Cohen’s F scores for radiomic features before and after ComBat harmonization in all 6 experiments. Of 172 radiomic features, 76.7% (Siemens), 52.9% (GE), and 26.7% (Philips) of features were significantly different between 1.5 T and 3 T in Experiments 1–3. Among three manufacturers at 1.5 T, 155/172 (90.1%) radiomic features were significantly different in Experiment 4. Among three system manufacturers at 3 T, 129/172 (75.0%) radiomic features were observed to be significantly different in Experiment 5. In Experiment 6, we found that 117/172 (68.0%) radiomic features were significantly different between systems from different manufacturers and field strengths. After ComBat harmonization, no significant difference was observed on radiomic features among manufacturers or field strengths. In all (6/6) experiments, Cohen’s F scores for radiomic features were reduced significantly after ComBat harmonization.

**Table 2:** The number of features with significant differences and Cohen’s F score for radiomic features before and after ComBat harmonization

Exp	Non-biological variations	Controlled variables	The number of features with significant differences (*p* < 0.05)		Mean Cohen’s F sensitivity (mean ± SD)	
			Original	Harmonized	Original	Harmonized
1	Field strengths (1.5 T vs 3 T)	Site 1 (Siemens)	132/172 (76.7%)	0/172 (0%)	0.256 ± 0.187	0.003 ± 0.002
2		Site 2 (GE)	91/172 (52.9%)	0/172 (0%)	0.141 ± 0.115	0.006 ± 0.005
3		Site 3 (Philips)	46/172 (26.7%)	0/172 (0%)	0.144 ± 0.085	0.020 ± 0.015
4	System manufacturers (Siemens vs GE vs Philips)	1.5 T	155/172 (90.1%)	0/172 (0%)	0.384 ± 0266	0.002 ± 0.001
5		3 T	129/172 (75.0%)	0/172 (0%)	0.245 ± 0.193	0.006 ± 0.004
6	Field strengths and manufacturers	N/A	117/172 (68.0%)	0/172 (0%)	0.162 ± 0.145	0.001 ± 0.001

**Fig. 2:**
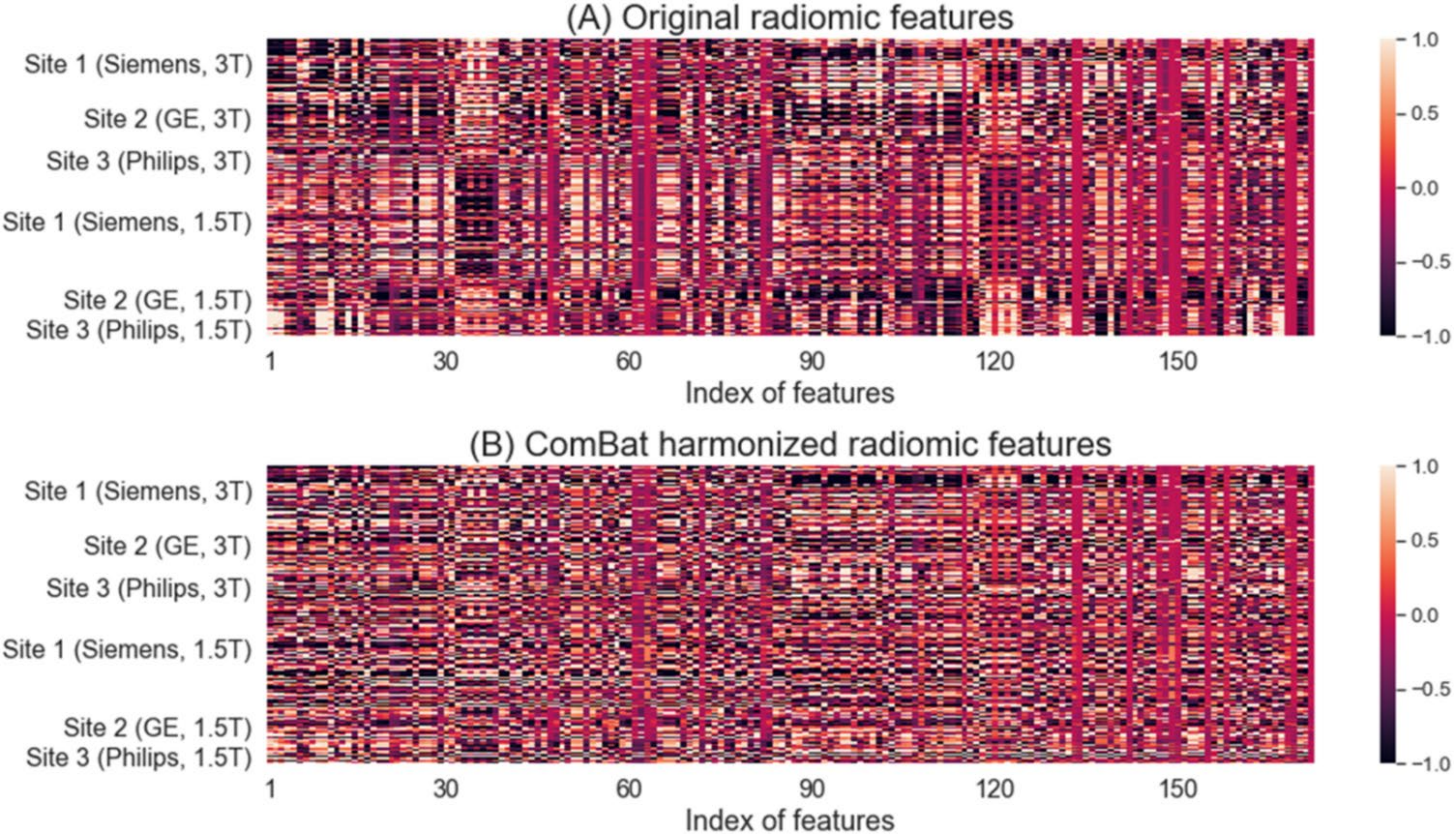
Heatmaps of radiomic features of all 3857 MRI exams in Experiment 6 before and after harmonization. **A** Original radiomic features and **B** ComBat harmonized radiomic features. Each row corresponds to individual T2W MRI exams, while each column corresponds to individual radiomic features. Z-score transformation was applied to normalize individual radiomic features. MRI exams are grouped based on study site (manufacturer, field strength)

To visualize the batch effects before and after ComBat harmonization, we generated heatmaps of z-score normalized radiomic features from all MRI exams in Experiment 6 (Fig. 2). The heatmap of original radiomic features shows distinct color variations across study sites (manufacturer, field strength), indicating that batch effects exist in radiomic features (Fig. 2A). In contrast, the heatmap of ComBat harmonized features exhibits uniform variability across patients from different groups (Fig. 2B). This suggests that the harmonization process was successful in mitigating batch effects.

We also separated liver and spleen radiomic features before performing all five experiments (Tables 3 and 4). Between different field strengths, the numbers of significantly different liver radiomic features with significant differences were 84/86 (97.7%) for Siemens, 33/86 (38.4%) for GE, and 31/86 (36.0%) for Philips, respectively. No significantly different radiomic features of the liver were found after the ComBat harmonization. The numbers of significantly different spleen radiomic features were 48/86 (55.8%) for Siemens, 58/86 (67.4%) for GE, and 15/86 (17.4%) for Philips, respectively. Similarly, no significantly different spleen radiomic features were found after the ComBat harmonization.

**Table 3:** The number of features with significant differences and Cohen’s F score for liver radiomic features before and after ComBat harmonization

Exp	Non-biological variations	Controlled variables	The number of features with significant differences (*p* < 0.05)		Mean Cohen’s F sensitivity (mean ± SD)	
			Original	Harmonized	Original	Harmonized
1	Field strengths (1.5 T vs 3 T)	Site 1 (Siemens)	84/86 (97.7%)	0/86 (0%)	0.351 ± 0.153	0.004 ± 0.002
2		Site 2 (GE)	33/86 (38.4%)	0/86 (0%)	0.107 ± 0.093	0.005 ± 0.004
3		Site 3 (Philips)	31/86 (36.0%)	0/86 (0%)	0.163 ± 0.095	0.025 ± 0.019
4	System manufacturers (Siemens vs GE vs Philips)	1.5 T	80/86 (93.0%)	0/86 (0%)	0.402 ± 0.224	0.002 ± 0.001
5		3 T	67/86 (77.9%)	0/86 (0%)	0.200 ± 0.128	0.007 ± 0.004
6	Field strengths and manufacturers	N/A	57/86 (66.3%)	0/86 (0%)	0.132 ± 0.130	0.001 ± 0.001

**Table 4:** The number of features with significant differences and Cohen’s F score for spleen radiomic features before and after ComBat harmonization

Exp	Non-biological variations	Controlled variables	The number of features with significant differences (*p* < 0.05)		Mean Cohen’s F sensitivity (mean ± SD)	
			Original	Harmonized	Original	Harmonized
1	Field strengths (1.5 T vs 3 T)	Site 1 (Siemens)	48/86 (55.8%)	0/86 (0%)	0.160 ± 0.160	0.002 ± 0.001
2		Site 2 (GE)	58/86 (67.4%)	0/86 (0%)	0.177 ± 0.123	0.008 ± 0.005
3		Site 3 (Philips)	15/86 (17.4%)	0/86 (0%)	0.128 ± 0.069	0.016 ± 0.009
4	System manufacturers (Siemens vs GE vs Philips)	1.5 T	75/86 (87.2%)	0/86 (0%)	0.363 ± 0.299	0.002 ± 0.001
5		3 T	62/86 (72.1%)	0/86 (0%)	0.291 ± 0.231	0.005 ± 0.004
6	Field strengths and manufacturers	N/A	60/86 (69.8%)	0/86 (0%)	0.190 ± 0.149	0.002 ± 0.001

For 1.5 T, 80/86 (90.3%) liver radiomic features were significantly different among the three different manufacturers, while for 3 T, 67/86 (77.9%) liver radiomic features were significantly different. Meanwhile, for 1.5 T, 75/86 (87.2%) spleen radiomic features were significantly different among three different manufacturers, while for 3 T, 62/86 (72.1%) spleen radiomic features were significantly different. In Experiment 6, 57/86 (66.3%) and 60/86 (69.8%) radiomic features were observed significantly different for the liver and spleen, respectively. No liver or spleen radiomic features were found significantly different after ComBat harmonization. Cohen’s F scores for both liver and spleen radiomic features decreased significantly after ComBat harmonization.

**Fig. 3:**
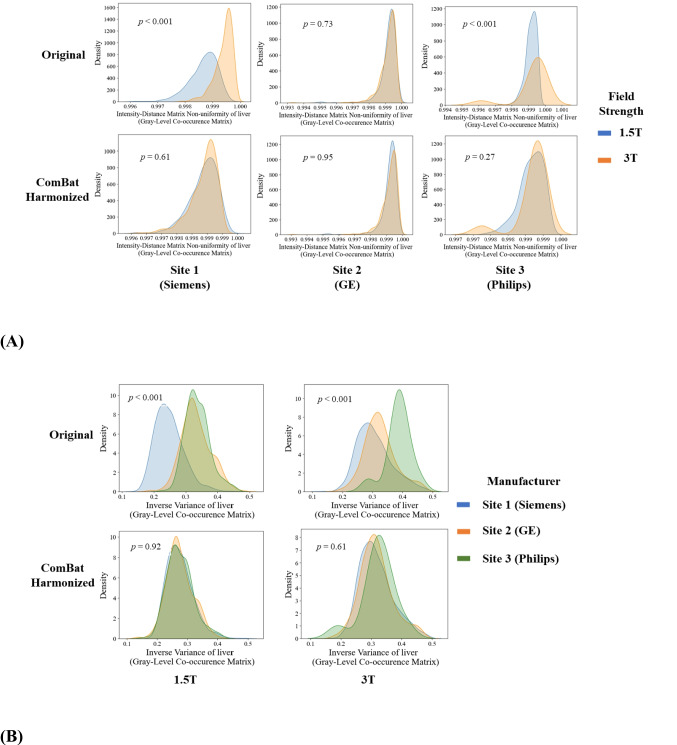
**A** Kernel density plots of intensity-distance matrix non-uniformity of liver by field strength. The top row is for the original data, and the bottom row is for ComBat harmonized data. Columns are plots for Site 1 (Siemens), Site 2 (GE), and Site 3 (Philips), from left to right.** B** Kernel density plots of inverse variance of the liver by system manufacturers. The top row is for the original data, and the bottom row is for ComBat harmonized data. The left column is for data from 1.5 T systems, and the right column is for data from 3 T systems

We visualized the distributions of several radiomic feature examples using kernel density plots before or after ComBat harmonization. Figure 3A illustrates distributions of intensity-distance matrix non-uniformity (IDMN) of the liver between 1.5 T and 3 T. Visually, the distributions of the original IDMN showed a distinct difference between 1.5 T and 3 T systems from Site 1 (Siemens) and Site 3 (Philips). Compared to this, harmonized IDMN exhibited very similar patterns between 1.5 T and 3 T across all sites. At Site 2 (GE), since the distributions of original IDMN were similar between 1.5 T and 3 T, ComBat had only a minimal adjustment on the distribution of IDMN. Figure 3B visualizes the distributions of inverse variance (IV) features of the liver across three manufacturers. By comparing the original and harmonized features, it is shown that ComBat was able to successfully eliminate distribution differences among the three manufacturers.

**Fig. 4:**
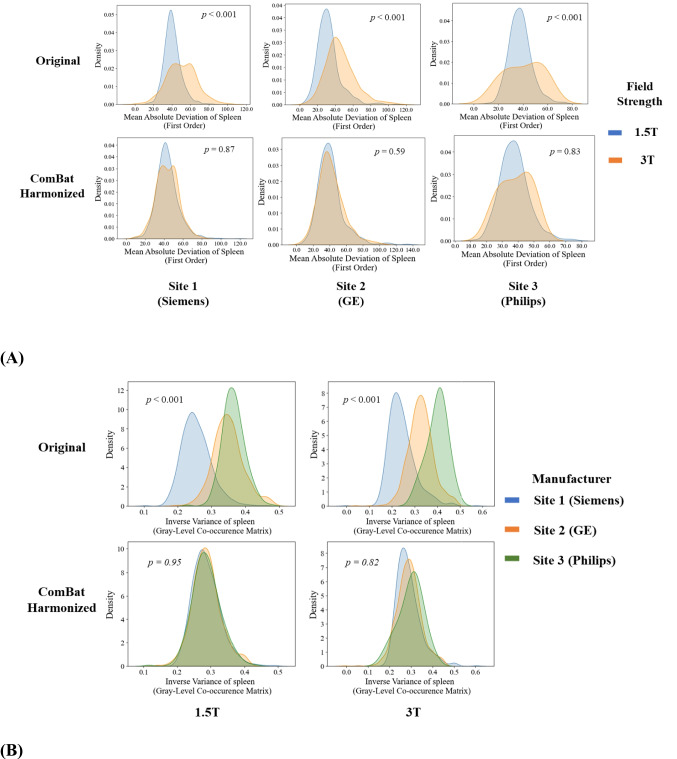
**A** Kernel density plots of mean absolute deviation of the spleen by field strengths. The top row is the original data, and the bottom row is ComBat harmonized data. Columns are plots for Site 1 (Siemens), Site 2 (GE), and Site 3 (Philips), from left to right. **B** Kernel density plots of inverse variance of the spleen by system manufacturers. The top row is for the original data, and the bottom row is for ComBat harmonized data. The left column is for data from 1.5 T systems, and the right column is for data from 3 T systems

The kernel density plots of the mean absolute deviation (MAD) of the spleen between 1.5 T and 3 T are shown in Fig. 4A. We observed that ComBat had successfully harmonized the distributions of MAD feature from Site 1 (Siemens) and Site 2 (GE). Apparently, ComBat was not as effective for Site 3 as for the other two sites. This is likely because the MAD feature of 3 T systems at Site 3 had a bimodal distribution in shape (i.e., two prominent peaks), which is much wider spread than MAD feature of the 1.5 T systems before harmonization, resulting in additional difficulties in aligning two distributions well. The kernel density plots of IV features of the spleen among three manufacturers are presented in Fig. 4B, where ComBat has successfully harmonized the distributions of IV features from Site 1 (Siemens), Site 2 (GE), and Site 3 (Philips). Visually, the distributions of spleen IV feature were perfectly aligned among different manufacturers’ 1.5 T systems, while these were only sub-optimally aligned on 3 T systems, even though significant differences cannot be detected among different manufacturers.

### Effectiveness of ComBat Harmonization on Deep Features

Table 5 lists the number of features with significant differences and Cohen’s F scores for deep features before and after ComBat harmonization in all 6 experiments. Of 1024 deep features, we observed that 89.0% (Siemens), 56.5% (GE), and 0.1% (Philips) of features have significant differences between 1.5 T and 3 T. Among the three manufacturers at 1.5 T, 914/1024 (89.3%) of deep features had significant differences. Among 3 manufacturers at 3 T, 861/1024 (84.1%) of deep features had significant differences. In Experiment 6, 858/1024 (83.8%) deep features were significantly different between systems from different manufacturers and field strengths. After ComBat harmonization, deep features among manufacturers or field strengths had no significant difference. Lower Cohen’s F scores were observed consistently for all (6/6) experiments after ComBat harmonization.

**Table 5:** The number of features with significant differences and Cohen’s F score for deep features before and after ComBat harmonization

Exp	Non-biological variations	Controlled variables	The number of features with significant differences (*p* < 0.05)		Mean Cohen’s F sensitivity (Mean ± SD)	
			Original	Harmonized	Original	Harmonized
1	Field strengths (1.5 T vs 3 T)	Site 1 (Siemens)	911/1024 (89.0%)	0/1024 (0%)	0.437 ± 0.311	0.001 ± 0.001
2		Site 2 (GE)	579/1024 (56.5%)	0/1024 (0%)	0.164 ± 0.119	0.007 ± 0.005
3		Site 3 (Philips)	2/1024 (0.1%)	0/1024 (0%)	0.059 ± 0.046	0.022 ± 0.018
4	System manufacturers (Siemens vs GE vs Philips)	1.5 T	914/1024 (89.3%)	0/1024 (0%)	0.483 ± 0.361	0.001 ± 0.001
5		3 T	861/1024 (84.1%)	0/1024 (0%)	0.387 ± 0.276	0.003 ± 0.002
6	Field strengths and manufacturers	N/A	858/1024 (83.8%)	0/1024 (0%)	0.227 ± 0.163	0.001 ± 0.001

**Fig. 5:**
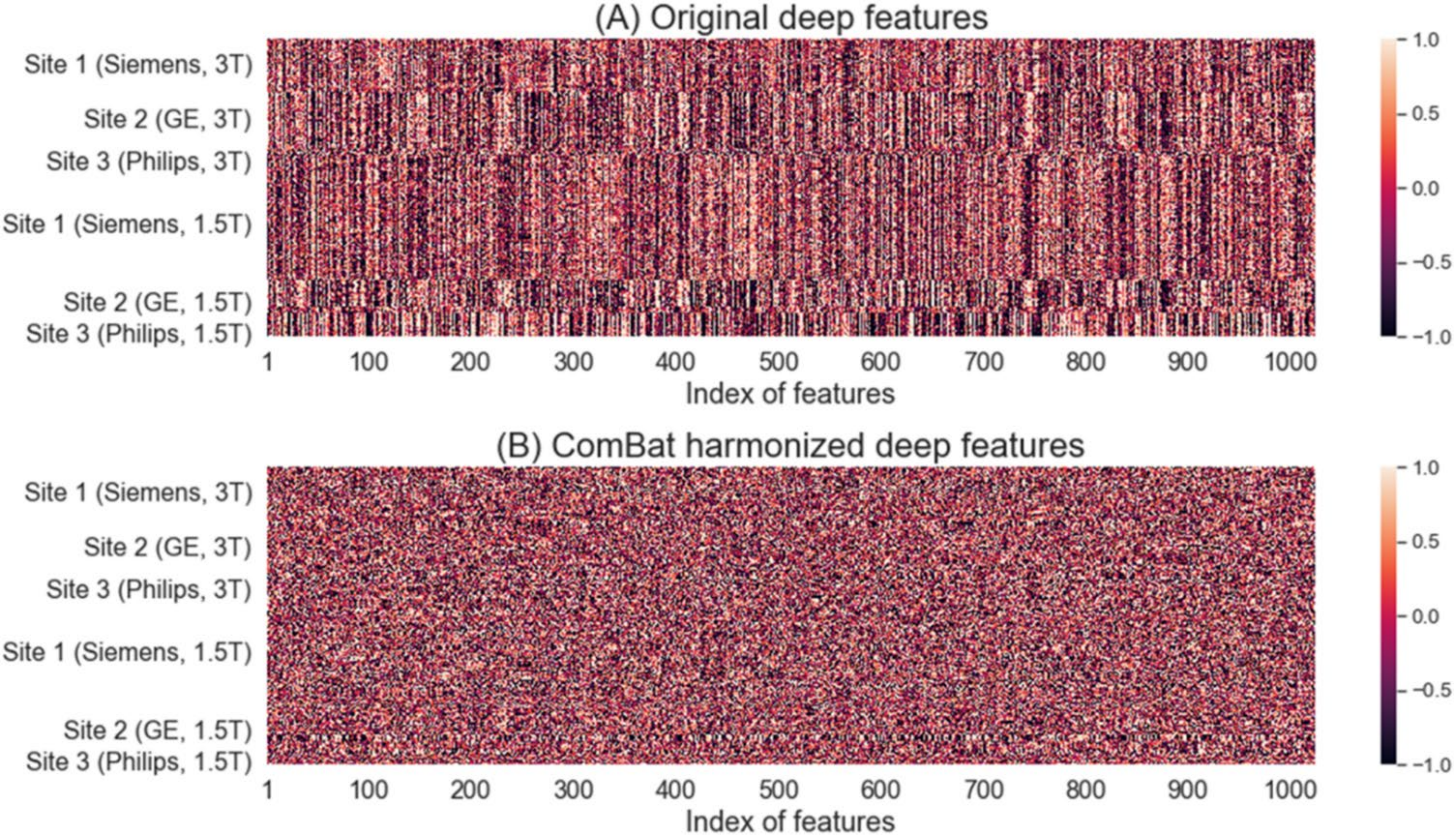
Heatmaps of deep features of all 3857 MRI exams in Experiment 6 before and after harmonization. **A** Original deep features and **B** ComBat harmonized deep features. Each row corresponds to individual T2W MRI exams, while each column corresponds to deep features. Z-score transformation was applied to normalize individual deep features. MRI exams are grouped based on study site (manufacturer, field strength)

We illustrated the batch effects in deep features before and after ComBat harmonization using heatmaps of deep features from all MRI exams in Experiment 6 (Fig. 5). Similar to radiomic features, we also applied z-score transformation to normalize individual deep features. Figure 5A shows the heatmap of original deep features, where distinct color variations exist across subjects from different study sites, manufacturer, and field strength. This suggests that there were batch effects in original deep features. The heatmap in Fig. 5B exhibits uniform variation across all MRI exams, indicating reduced batch effects among deep features.

**Fig. 6:**
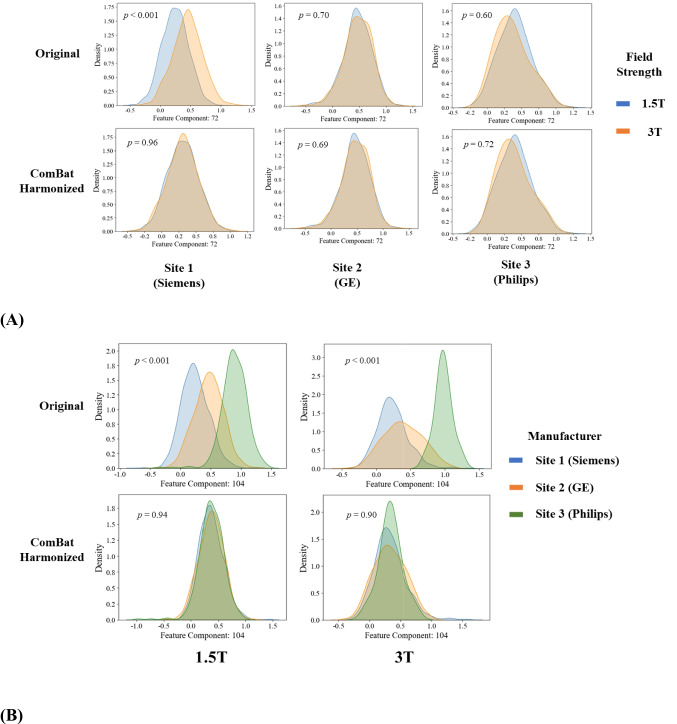
**A** Kernel density plots of deep feature (component index = 72) distributions by field strengths. The top row is the original data, and the bottom row is ComBat harmonized data. Columns are plots for Site 1 (Siemens), Site 2 (GE), and Site 3 (Philips), from left to right. **B** Kernel density plots deep feature (component index = 104) distributions by system manufacturers. The top row is for the original data, and the bottom row is for ComBat harmonized data. The left column is for data from 1.5 T systems, and the right column is for data from 3 T systems

The distributions of deep features before or after ComBat harmonization were also visualized using kernel density plots. Figure 6A illustrates distributions of a random deep feature (component index = 72) between 1.5 T and 3 T. The component index was randomly selected from 1024 components of deep features from Swin Transformer. The original features from Site 1 (Siemens) had a distinct difference between 1.5 T and 3 T systems, while the original features from Site 2 (GE) and Site 3 (Philips) had no significant difference between field strengths. After harmonization, this deep feature (component index = 72) at Site 1 (Siemens) had very similar patterns between 1.5 T and 3 T. Figure 6B shows another random deep feature (component index = 104) across three manufacturers. We noted that ComBat can also successfully harmonize distribution among three manufacturers with the same field strengths.

## Discussion

Our study investigated whether ComBat harmonization can effectively remove non-biological variations due to either field strengths and/or manufacturers from radiomic and deep features using a large, multi-center clinical abdominal T2W MRI data. The results validate our hypothesis that ComBat can effectively harmonize radiomic and deep features from different MRI systems of different field strengths and/or manufacturers. Previously, Priya et al. investigated the effect of ComBat harmonization on the robustness of cardiac MRI-derived radiomic features to variations in imaging parameters and found that the stability of radiomic features to changes in imaging parameters across all cardiac MRI was significantly improved [27]. In another recent work [28], Leithner et al. demonstrated that the ComBat harmonization is able to improve multiclass radiomics-based tissue classification using T1-weighted 3D gradient echo Dixon MRI from a cohort of 100 subjects at two study centers. We observed similar results to those recent works in our current investigation. Our study extends this line of research by investigating both radiomic and deep features for abdominal MRI data from a large cohort of exams from a variety of MRI systems with different field strengths and from three major MRI manufacturers. Such a large dataset allows us to rigorously determine the effectiveness of the ComBat method with great statistical power and to draw firm conclusions.

Using MRI data from multiple study sites can substantially increase the rigor of scientific investigations [36]. By involving multiple centers, researchers can recruit a larger and more diverse group of participants, significantly increasing the statistical power of the study and improving the generalizability of the findings [37, 38]. Despite many advantages, differences in imaging protocols within or between study sites can affect the homogeneity of extracted features in multi-center MRI studies due to non-biological variations. Non-biological variation in MRI data refers to sources of variability in MRI scans that are not related to the underlying biology or physiology of the subject being imaged. Common non-biological sources include system manufacturers, field strength, gradient coil performance, or RF coil sensitivity. Non-biological variations can be reflected in intensity distributions, which can further affect distributions of extracted features. For all downstream classification tasks using features extracted from multi-center data, model performance and overall generalizability could be compromised [21, 38]. Therefore, harmonization is crucial to multi-center MRI studies to remove potential batch effects [24, 39, 40].

Harmonization has been utilized to facilitate various multi-center medical image analyses [23]. Previous studies have shown that harmonization can be applied to improve the performance of AI-powered diagnosis or the identification of biomarkers [41–43]. Among different harmonization techniques, ComBat serves as an effective and computationally efficient method that can be applied to any medical image features. ComBat exhibits several advantages, such as no need for preliminary phantom experiments or test sets before use and the ability to use previously extracted features [44]. These make ComBat easy to integrate into feature analysis pipelines. More importantly, ComBat does not modify or remove biological variants of image features by involving biological covariates (e.g., sex and age) into the modeling process, thus safeguarding biologically or clinically meaningful information [45, 46]. By employing empirical Bayesian models for estimating transformations specific to imaging parameters, ComBat effectively eliminates non-biological batch-related effects [45]. ComBat has its own limitations. While ComBat is effective on harmonizing features of medical images, it was not designed to harmonize original 2D or 3D medical images directly. For example, Kushol et al. found that harmonized images generated by the ComBat method fail to enhance the classification accuracy in most of their brain MR image classification applications [42]. Thus, the ComBat method should be predominantly limited to feature-level harmonization, as opposed to pixel-level harmonization for the entire MR image.

This study has limitations. First, we used only T2W MRI data in the current investigation. However, we anticipate that our results will be generalizable to other MR contrasts (e.g., T1-weighted and diffusion-weighted images). Second, our study assessed only the performance of ComBat on segmented liver and spleen for radiomic features as well as unsegmented upper abdomen for deep features. This was partly due to the availability of a robust and automated organ segmentation model. In future studies, other organs of interest can be studied when automated segmentation strategies or manual segmentations become available. Finally, we used only two of numerous potential state-of-the-art strategies for extracting features—PyRadiomics for radiomic feature extraction and Swin Transformer for deep feature extraction.

In summary, we have shown that ComBat can be used to harmonize the radiomic and deep features of T2W MR images from a large multi-center, multi-vendor MRI dataset. Such harmonization is important to remove non-biological variations due to different field strengths or vendors. Our findings support the use of ComBat for dataset harmonization in multi-center research or intra-institutional research in the setting of multiple MRI systems.

## Data Availability

The data that support the findings of this study are not openly available due to reasons of sensitivity. Data are located in controlled access data storage at Cincinnati Children's Hospital Medical Center.

## Abbreviations

AI: Artificial intelligence
IDMN: Intensity-distance matrix non-uniformity
IV: Inverse variance
MAD: Mean absolute deviation
MRI: Magnetic resonance imaging
